# Correlation Analysis of Plasma Myeloperoxidase Level With Global Registry of Acute Coronary Events Score and Prognosis in Patients With Acute Non-ST-Segment Elevation Myocardial Infarction

**DOI:** 10.3389/fmed.2022.828174

**Published:** 2022-03-28

**Authors:** Nan Zhang, Jing-Xian Wang, Xiao-Yuan Wu, Yan Cui, Zhong-He Zou, Yin Liu, Jing Gao

**Affiliations:** ^1^Thoracic Clinical College, Tianjin Medical University, Tianjin, China; ^2^Department of Cardiology, Tianjin Chest Hospital, Tianjin, China; ^3^Cardiovascular Institute, Tianjin Chest Hospital, Tianjin, China

**Keywords:** myeloperoxidase, NSTEMI, prognosis, MACE, GRACE score

## Abstract

**Background:**

Myeloperoxidase (MPO) and global registry of acute coronary events (GRACE) risk scores were independently used to predict adverse outcomes in patients with acute coronary syndrome (ACS). However, the relationship between MPO level and GRACE score, and whether the combination of MPO and GRACE can better predict major adverse cardiovascular events (MACEs) in patients with acute non-ST-segment elevation myocardial infarction (NSTEMI), have not been previously investigated.

**Methods:**

A prospective cohort of 271 consecutive patients with NSTEMI were enrolled in this study. Plasma MPO levels were measured by ELISA. Baseline demographic and clinical information was collected, and GRACE scores were calculated at admission. The correlation between MPO and MACEs was evaluated with the GRACE score during a 1-year follow-up.

**Results:**

The results showed that plasma MPO level was correlated with inflammatory indices (including high-sensitivity C-reactive protein (hs-CRP), leukocyte count, neutrophil count, and fibrinogen), N-terminal pro-B type natriuretic peptide (NT-proBNP), and hypersensitive troponin T (hsTNT) levels (All *p*-values < 0.05), and there was a statistically significant correlation between plasma MPO level and GRACE score (*r* = 0.22, *p* < 0.001). The Kaplan-Meier curves showed that patients with higher MPO levels had lower event-free survival (Log-rank *P* < 0.001). The multivariate Cox model showed MPO was an independent risk factor for 1-year MACEs in patients with NSTEMI (HR: 3.85, 95% CI: 1.4–10.6, *p* = 0.009). Subgroup analysis showed that MPO was a strong prognostic biomarker, and its prognostic value was more significant in patients with age >65 years and N-terminal pro-B type natriuretic peptide (NT-proBNP) level >1,000 pg/ml. For high-risk patients with GRACE scores, a higher level of MPO has a higher prognostic value.

**Conclusion:**

Elevated plasma MPO levels are associated with high inflammatory status and GRACE scores in patients with NSTEMI. For high-risk patients with GRACE scores, higher MPO levels were more predictive of future MACEs.

## Introduction

In recent years, the incidence of non-ST segment elevation myocardial infarction (NSTEMI) is on the rise, gradually exceeding that of ST-segment elevation myocardial infarction (STEMI) (1–3). Patients with NSTEMI have more comorbidities and a higher prevalence of multiple coronary artery diseases (CADs), and the long-term prognosis of the patients is poor (4). Some patients still have major adverse cardiovascular events (MACEs) after discharge (5). Therefore, how to assess and predict the occurrence of MACEs as early as possible has become a key topic of clinical research, which is of great clinical significance for early intervention measures to improve the prognosis.

Most acute coronary syndromes (ACS) have two mechanisms: plaque fibrous cap rupture and intimal surface erosion. Inflammation plays an important role in the occurrence and development of ACS. It can mediate the weakening of the fibrous cap of atherosclerotic plaque and make plaque rupture more easily, thus inducing ACS (6). Many studies have shown that inflammatory biomarkers can predict the future risk and adverse clinical outcome of NSTEMI. Among all the inflammatory biomarkers, myeloperoxidase (MPO) is secreted by inflammatory cells and catalyzes various oxidative reactions, which is one of the markers of inflammation and oxidative stress. It participates in almost all stages of atherosclerosis, from endothelial dysfunction, oxidative modification of LDL and HDL to plaque instability and vulnerability, increasing the sensitivity of NSTEMI diagnosis (7–9). Early clinical studies have also shown that MPO levels strongly predict the increased risk of subsequent cardiovascular events in patients with NSTEMI, expanding prognostic information from traditional biochemical markers such as cardiac troponin and brain natriuretic peptide (10, 11). However, the value of MPO for risk stratification in patients with NSTEMI has not been widely studied.

The Global Registry of Acute Coronary Events (GRACE) scoring system is a predictive tool for risk stratification of patients with NSTEMI. However, biomarkers with pathophysiological mechanisms are not included. Therefore, this study intends to investigate the relationship between plasma MPO level and GRACE score, and whether the combination of the two can better predict the occurrence of MACEs in patients with NSTEMI, to provide further reference data for judging the disease and choosing treatment options.

## Methods

### Study Population

We recruited 271 patients diagnosed with NSTEMI from January 2016 to December 2017 in Tianjin Chest Hospital. The inclusion criteria were: all participants who underwent clinical evaluation, including a history of chest pain, physical examination, a series of serum cardiac biomarker tests, 12-lead electrocardiogram, and coronary angiography. Participants who met the European Society of Cardiology/American College of Cardiology NSTEMI diagnostic criteria: troponin and/or CK-MB exceeded the reference value by 99% at the baseline, and at least one of them was associated with: (1) ST-segment depression or T-wave inversion; (2) with chest pain symptoms lasting more than 30 min (12, 13) were also included. The exclusion criteria were severe infectious or immune diseases, other malignancies, severe liver or kidney dysfunction, serious heart disease other than coronary heart disease, cognitive or communication disorders, and patients who refused to participate in the study at any stage.

All patients and their families agreed to participate in the study and signed informed consent forms after being informed of the procedures and risks involved. The study was carried out in accordance with the Declaration of Helsinki. This study had been approved by the ethical committee of Tianjin Chest Hospital. This study was registered at ClinicalTrials.gov (identifier NCT03600259).

### Sample Collection and Laboratory Testing

Patients fasted overnight on the day of admission (≥8 h), and blood samples were taken in the morning. For the patients whose GRACE score was rated as high-risk group (>140) and who needed emergency intervention, the sampling time was before the intervention. The blood sample was stored at 4°C, centrifuged for 10 min at 3,000 rpm for 2 h after collection, and then stored at -80°C until measurement. Plasma MPO levels were determined by ELISA using a commercial kit (R&D Systems, Minneapolis, MN, United States).

### Data Collection

Demographic data, characteristics of admission, complications, treatment modalities, medications, and laboratory tests were recorded. Eight variables were used to calculate GRACE scores for all patients at admission, including age, systolic blood pressure, heart rate, serum creatinine, Killip grade, cardiac arrest at admission, elevated cardiac injury biomarker, and ST-segment offset. The total score of the GRACE scale was 372. A score >140 is considered high risk; 109–140 is medium risk; <108 is classified as low risk.

### Study Endpoints

A total of 271 NSTEMI patients were followed up for 1 year. Patients were followed up by case review, telephone follow-up, or outpatient follow-up by a trained nurse or physician until the last day of the follow-up period, or the occurrence of MACEs. The study endpoints were MACEs, which included all-cause death, non-fatal recurrent myocardial infarction (MI), target lesion revascularization (TLR) and hospital admission for heart failure (HF). Non-fatal MI was defined by typical angina of ≥ 20 min duration, ECG changes, and a rise in troponin or creatine kinase level above the 99 percentile of the upper reference limit. TLR refers to revascularization [percutaneous coronary intervention (PCI) or coronary artery bypass grafting (CABG)] due to ischemic symptoms or objective journal evidence and target stenosis >50%.

### Statistical Methods

Plasma MPO levels were classified into three levels: low, medium, and high using an X-tile procedure (Yale University, New Haven, CT, United States). The X-tile plots can provide a single, global assessment of every possible way of dividing a cohort into low-, medium-and high-level marker expressions. Moreover, this project gives rise to a rigorously statistical estimation by dividing a single cohort into training and validation subsets for the best *P*-value evaluation when separate training and validation cohorts are not available. The X-tile analysis considers variables and patient survival when selecting cut-off points to ensure homogeneity within and heterogeneity between groups as much as possible (14).

Continuous data are expressed as mean ± standard deviation or median (quartile range). Categorical variables are expressed as counts (percentages). The *T*-test, one-way ANOVA, and the Kruskal-Wallis H test were used to evaluate the differences in continuous variables between groups. The chi-square test was used for categorical variable comparison, and Fisher’s exact probability method was used when the theoretical frequency of counting data was less than 5. Multiple comparisons were performed using the Bonferroni method. Patients were divided into different groups based on plasma MPO levels. The Kaplan-Meier curve used a logarithmic rank test to estimate the event-free survival rate between groups. The univariate and multivariate Cox proportional risk models were used to determine the major impact of MPO levels on the above adverse outcomes. Exploratory subgroup analysis was performed based on expertise. SPSS 26.0 software was used for data processing, and *P* < 0.05 was considered as a statistically significant difference.

## Results

### Myeloperoxidase Cut-Off Point Selection

The optimal cut-off points for plasma MPO determined by the X-tile are: low level (6.89–49.17 ng/ml), medium level (49.17–85.47 ng/ml), and high level (85.47–241.45 ng/ml) (Table 1 and Supplementary Figure 1).

**TABLE 1:** Cut-off point of MPO by X-tile method.

Group	Range (ng/mL)	Patient	Percent (%)	Event
Low	6.89–49.17	129	47.6	9
Medium	49.17–85.47	86	31.73	12
High	85.47–241.45	56	20.66	15
Total	6.89–241.45	271	100	36

### Myeloperoxidase and Baseline Characteristics

Baseline characteristics of 271 NSTEMI patients were stratified based on plasma MPO levels (Table 2). There were 188 men (69.37%) and 83 women (30.63%). The older the age, the higher the MPO level (*p* = 0.012). There was a negative correlation between MPO level and LVEF (*p* = 0.01). The most common history was hyperlipidemia in 208 cases (77.9%), followed by hypertension in 176 cases (64.94%), current/former smoking in 155 cases (57.20%), diabetes in 91 cases (33.58%), stroke in 55 cases (20.30%), and family history in 28 cases (10.33%). Among 271 patients, 48 (22.02%) had a single-vessel disease and 166 (76.15%) had a multi-vessel disease. A total of 90 patients (33.21%) chose medication treatment, 162 patients (59.78%) underwent PTCA/PCI, and 19 patients (7.01%) underwent CABG. According to patients’ clinical characteristics and doctors’ judgment, 268 patients (98.89%) received DAPT, 227 (83.76%) received beta-blockers, 164 (60.52%) received ACEI/ARB, 270 (99.63%) received anticoagulant, and 227 (83.76%) received statin. There were no significant differences in IABP application, breathing machine, heart rate, systolic pressure, diastolic pressure, history, Killip classification, revascularization strategy, type of CAD, and medication during the follow-up in groups with high, medium, and low MPOs (All values of *p* > 0.05).

**TABLE 2:** Baseline characteristics of the NSTEMI patients in different MPO level.

Characteristics	Overall (*N* = 271)	Low (*N* = 129)	Medium (*N* = 86)	High (*N* = 56)	*p*-value
Age, years	65 (57, 73)	62 (54.75, 71.25)	65 (59.5, 74)	67 (62.5, 76.25)	0.012
Male, n (%)	188 (69.37)	99 (76.74)	61 (70.93)	28 (50.00)	0.001
IABP, n (%)	15 (5.54)	8 (6.20)	5 (5.81)	2 (3.57)	0.884
Breathing machine, n (%)	36 (13.3)	19 (14.7)	9 (10.50)	8 (14.3)	0.671
Heart rate	74 (63, 84)	72 (62, 84.25)	72 (63.5, 83.25)	78 (65, 84.25)	0.309
Systolic pressure, mmHg	133.07 ± 21.55	134.56 ± 23.11	132.43 ± 20.54	130.61 ± 19.31	0.493
Diastolic pressure, mmHg	75 (69, 85)	75 (67.75, 86.25)	76 (70, 85)	75 (67, 82)	0.597
LVEF, n (%)	54 (47, 57)	55 (48.5, 58.75)	55 (50, 57)	48 (36, 52.5)	0.010
**Past history**					
Current/former smoking, n (%)	155 (57.20)	81 (62.79)	47 (54.65)	27 (48.21)	0.153
Hypertension, n (%)	176 (64.94)	78 (60.47)	58 (67.44)	40 (71.43)	0.304
Diabetes, n (%)	91 (33.58)	45 (34.88)	28 (32.56)	18 (32.14)	0.902
Previous stroke, n (%)	55 (20.30)	21 (16.28)	20 (23.26)	14 (25.00)	0.281
Previous MI, n (%)	37 (13.65)	19 (14.73)	10 (11.63)	8 (14.29)	0.825
Hyperlipidemia, n (%)	208 (77.90)	103 (80.47)	67 (79.76)	38 (69.09)	0.214
Family history of CAD, n (%)	28 (10.33)	16 (12.40)	8 (9.30)	4 (7.14)	0.529
**Biochemical characteristics**					
TG, mmol/L	1.61 (1.2, 2.25)	1.63 (1.23, 2.32)	1.59 (1.13, 2.23)	1.61 (1.23, 2.13)	0.519
TC, mmol/L	4.58 (3.76, 5.19)	4.49 (3.74, 5.12)	4.61 (3.92, 5.23)	4.61 (3.56, 5.2)	0.882
HDL-C, mmol/L	0.97 (0.85, 1.16)	0.96 (0.82, 1.14)	0.98 (0.85, 1.15)	0.99 (0.85, 1.19)	0.694
LDL-C, mmol/L	3.03 (2.3, 3.58)	3.09 (2.35, 3.68)	2.88 (2.38, 3.59)	2.84 (2.17, 3.25)	0.545
ApoA, g/L	1.13 (1.03, 1.27)	1.13 (1.02, 1.29)	1.12 (1.03, 1.24)	1.14 (0.98, 1.28)	0.893
ApoB, g/L	1.08 (0.87, 1.31)	1.06 (0.85, 1.31)	1.11 (0.95, 1.3)	1.11 (0.87, 1.33)	0.702
Lp(a), nmol/L	62.6 (22.18, 129.23)	67.5 (17.05, 136.2)	57.4 (26.75, 128.75)	65.35 (25.78, 113.1)	0.906
D-Dimer, μg/mL	0.43 (0.28, 0.72)	0.42 (0.29, 0.6)	0.39 (0.27, 1.02)	0.5 (0.32, 1.03)	0.15
Cr, μmol/L	77 (65.75, 95.25)	77 (65.75, 95.25)	77 (67.75, 95.25)	79 (69, 94.75)	0.621
hsTNT, ng/mL	0.54 (0.24, 1.39)	0.49 (0.21, 1.06)	0.52 (0.23, 1.16)	0.91 (0.4, 2.1)	0.013
hs-CRP, mg/L	5.27 (2.53, 14.74)	4.29 (1.83, 10.67)	5.26 (2.76, 19.83)	10.27 (4.02, 55)	0.001
leukocyte count,/10^9^	8.25 (6.7, 9.91)	7.71 (6.69, 9.58)	8.36 (6.51, 9.48)	9.58 (3.63, 32.99)	<0.001
Neutrophil count,/10^9^	5.93 (4.59, 7.65)	5.55 (4.5, 7.01)	5.83 (4.5, 7.44)	7.44 (5.66, 9.36)	<0.001
NT-proBNP, pg/ml	864.15 (324.13, 2356.75)	719.85 (219.18, 1688.25)	821.95 (368.68, 2462.25)	2116 (575.28, 4537.75)	0.001
Fibrinogen, g/L	3.59 (3.02, 4.32)	3.4 (2.8, 3.93)	3.62 (3.05, 4.3)	4.46 (3.56, 5)	<0.001
**Killip class**					
Killip I, n (%)	222 (81.92)	109 (84.50)	73 (84.88)	40 (71.43)	
Killip II-IV, n (%)	49 (18.08)	20 (15.50)	13 (15.12)	16 (28.57)	
**GRACE score, n (%)**					
<109	85 (31.37)	52 (40.31)	25 (29.07)	8 (14.29)	
109–140	103 (38.01)	47 (36.43)	36 (41.86)	20 (35.71)	
>140	83 (30.63)	30 (23.26)	25 (29.07)	28 (50.00)	
**Revascularization strategy, n (%)**					0.307
MT	90 (33.21)	38 (29.46)	27 (31.40)	25 (27.78)	
PTCA/PCI	162 (59.78)	83 (64.34)	52 (60.47)	27 (48.21)	
CABG	19 (7.01)	8 (6.20)	7 (8.14)	4 (7.14)	
**Type of coronary artery disease, n (%)**					0.647
No lesions	4 (1.83)	2 (1.82)	1 (1.43)	1 (2.63)	
Single-vessel lesion	48 (22.02)	23 (20.91)	19 (27.14)	6 (15.79)	
Multi-vessel lesion	166 (76.15)	85 (77.27)	50 (71.43)	32 (81.58)	
**Medication during follow-up, n (%)**					
DAPT	268 (98.89)	129 (100.00)	84 (97.67)	55 (98.21)	0.203
Beta-blocker	227 (83.76)	109 (84.50)	69 (80.23)	49 (87.50)	0.496
ACEI/ARB	164 (60.52)	78 (60.47)	54 (62.79)	32 (57.14)	0.802
Anticoagulant	270 (99.63)	129 (100.00)	85 (98.84)	56 (100.00)	0.524
Statin	265 (97.79)	126 (97.67)	86 (100.00)	53 (94.64)	0.072
**Outcome, n (%)**					
Composite MACEs	36 (13.28)	9 (6.98)	12 (13.95)	15 (26.79)	0.001
All-cause death	8 (2.95)	0	3 (3.49)	5 (8.93)	0.003
Non-fatal recurrent MI	8 (2.95)	3 (2.33)	4 (4.65)	1 (1.79)	0.637
Target lesion revascularization	14 (5.17)	4 (3.10)	3 (3.49)	7 (12.50)	0.03
Hospital admission for HF	11 (4.06)	2 (1.55)	5 (5.81)	4 (7.14)	0.096

*IABP, Intra-aortic balloon pump; LVEF, left ventricular ejection fraction; MI, Myocardial infarction; CAD, coronary artery disease; TG, triglyceride; TC, total cholesterol; HDL-C, high-density lipoprotein cholesterol; LDL-C, low-density lipoprotein cholesterol; ApoA, apolipoprotein A; ApoB, apolipoprotein B; Lp-a, Lipoprotein a; Cr, creatinine; hs-CRP, high-sensitivity C-reactive protein; NT-proBNP, N-terminal pro-B type natriuretic peptide; hsTNT, hypersensitive troponin T; GRACE, Global Registry of Acute Coronary Events; MT, medical therapy; PCI, percutaneous coronary Intervention; CABG, coronary artery bypass grafting; DAPT, dual antiplatelet therapy; ACEI, angiotensin-converting enzyme inhibitors; ARB, angiotensin II receptor blockers; Composite MACEs, consisted of all-cause death, non-fatal recurrent MI, target lesion revascularization, hospital admission for heart failure.*

### Myeloperoxidase and Other Biomarkers

**FIGURE 1:**
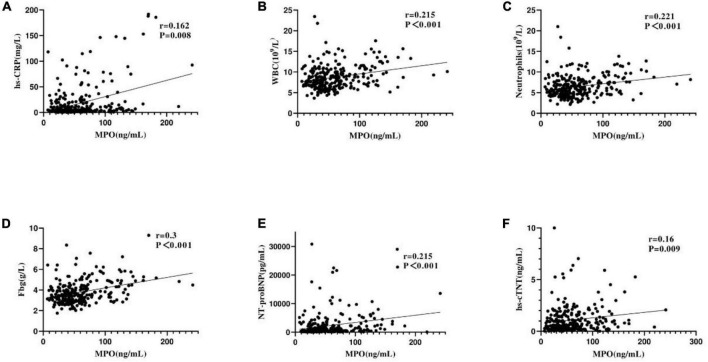
Correlation between plasma MPO levels and other biomarkers. Panels **(A–F)** represent the correlation between plasma MPO levels and high-sensitivity C-reactive protein (hs-CRP), white blood cell (WBC) count, neutrophils, fibrinogen(Fbg), NT-proBNP, and hypersensitive troponin T (hs-cTNT).

Spearman rank correlation analysis showed that in patients with NSTEMI, plasma MPO level was correlated with high-sensitivity C-reactive protein (hs-CRP) (*r* = 0.162, *p* = 0.008), leukocyte count (R = 0.215, *p* < 0.001), neutrophil count (*r* = 0.221, *p* < 0.001), fibrinogen (*r* = 0.3, *p* < 0.001), NT-proBNP (*r* = 0.215), *p* < 0.001), and hypersensitive troponin T (hsTNT) (*r* = 0.16, *p* = 0.009) (Figure 1). However, plasma MPO level was not correlated with serum lipid, D-Dimer, and creatinine levels, and serum lipid included TG, TC, HDL-C, LDL-C, ApoA, ApoB, and Lp(a) (all values of *P* > 0.05) (Table 2).

### Follow-Up and Results

A total of 271 patients with NSTEMI were followed up for 1 year, of which 11 were lost to follow-up. A total of 36 (13.28%) patients with NSTEMI had MACE events, all-cause death in eight cases (2.95%), non-fatal recurrent MI in eight cases (2.95%), target lesion revascularization in 14 cases (5.17%), and hospital admission for heart failure in 11 cases (4.06%). The incidence of MACEs increased significantly with low, medium, and high MPO levels (6.98 vs. 13.95 vs. 26.79%, *P* = 0.001). In addition, all-cause death showed a similarly significant upward trend (0 vs. 3.49 vs. 8.93%, *P* = 0.003). The incidence of target lesion revascularization was higher in the high MPO group than in the low or medium MPO groups (12.5 vs. 3.49 vs. 3.10%, *P* = 0.03). The incidence of no-fatal recurrent MI and hospital admission for HF was not statistically significant with MPO levels (Table 2).

### Prognostic Value of Myeloperoxidase

**FIGURE 2:**
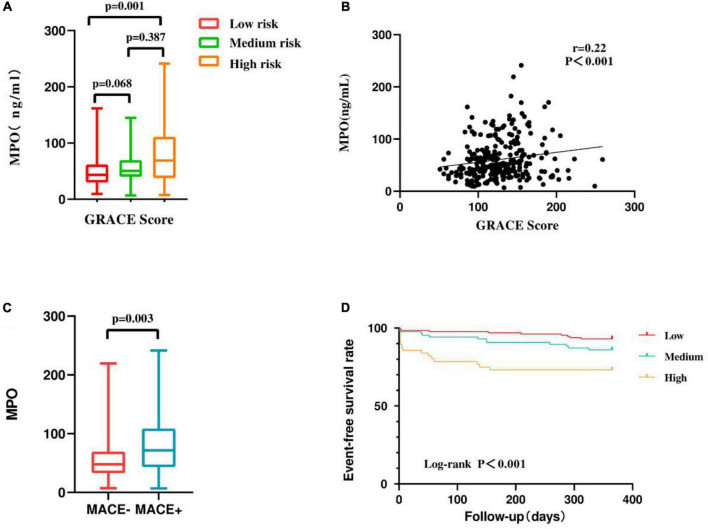
**(A)** Comparison of plasma MPO levels among groups based on GRACE score. **(B)** Correlation between GRACE score and plasma MPO levels. **(C)** Comparison of plasma MPO levels among groups based on MACEs. **(D)** Kaplan-Meier survival curve of 1-year MACEs in 271 NSTEMI patients. Model 1: adjustment for traditional cardiovascular risk factors (age, sex, current/former smoking, hypertension, hyperlipidemia, diabetes, previous stroke, and previous MI and family history of CHD). Model 2: Model 1 + Inflammation Marker (hs-CRP; leukocyte count; neutrophil count; fibrinogen). Model 3: Model 2 + GRACE score.

We found that MPO levels were higher in patients with MACEs than in patients without MACEs (*P* = 0.003) (Figure 2C). The Kaplan-Meier curves stratified by MPO levels showed a cumulative event-free survival rate during the 1-year follow-up. MPO levels were significantly associated with 1-year MACE events, and patients with higher MPO levels had a lower event-free survival rate (Log-rank *P* < 0.001) (Figure 2D).

**FIGURE 3:**
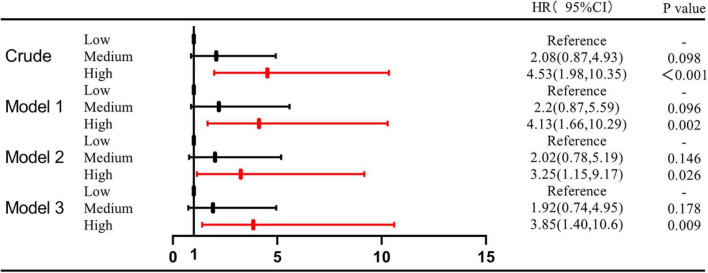
Hazard ratio for MACEs associated with plasma MPO levels in different models.

**FIGURE 4:**
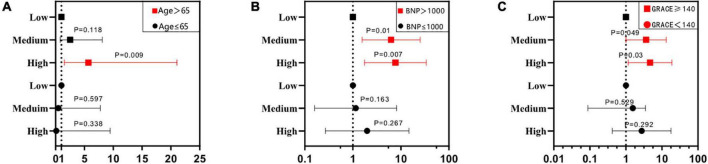
Subgroup analysis of different patients. Panels **(A–C)** represent the subgroups across different ages, NT-pro BNP levels, and GRACE scores. The red line denotes a statistically significant subgroup.

The univariate Cox regression analysis showed that a high level of MPO was associated with the occurrence of composite MACEs (medium level vs. low level, HR: 2.08, 95%CI: 0.87–4.93, *P* = 0.098; high level vs. low level, HR: 4.53, 95%CI: 1.98–10.35, *P* < 0.001). The multivariate Cox regression analysis showed that after adjusting for traditional cardiovascular risk factors [including age, sex, current/former smoking, hypertension, hyperlipidemia, diabetes, previous stroke, and previous MI and family history of coronary heart disease (CHD)], there was statistical significance between the high level of MPO and composite MACEs (Model 1: medium level vs. low level, HR: 2.2, 95%CI: 0.87–5.59, *p* = 0.096; high level vs. low level, HR: 4.13, 95%CI: 1.66–10.29, *P* = 0.002). When inflammatory markers were added to Model 1, the results were similar (Model 2: medium level vs. low level, HR: 2.02, 95%CI: 0.78–5.19, *p* = 0.146; high level vs. low level, HR: 3.25, 95%CI: 1.15–9.17, *P* = 0.026). Finally, we added GRACE scores to Model 2, which still maintained the correlation between high MPO levels and composite MACEs (Model 3: medium level vs. low level, HR: 1.92, 95%CI: 0.74–4.95, *p* = 0.178; high level vs. low level, HR: 3.85, 95%CI: 1.4–10.6, *P* = 0.009) (Supplementary Table 1 and Figure 3). In addition, the multifactor Cox model was used for subgroup analysis of composite MACEs at different MPO levels. We found that high levels of MPO were a strong prognostic biomarker for patients over 65 (medium level vs. low level, adjusted HR: 2.53, 95%CI: 0.79–8.09, *p* = 0.118; high level vs. low level, adjusted HR: 5.7, 95%CI: 1.54–21.05, *P* = 0.009). In patients with NT-proBNP > 1,000 pg/ml, moderate to high levels of MPO were a strong prognostic biomarker (medium level vs. low level, adjusted HR: 6.26, 95%CI: 1.56–25.12, *p* = 0.01; high level vs. low level, adjusted HR: 7.69, 95%CI: 1.75–33.71, *P* = 0.007) (Supplementary Table 2 and Figure 4).

The global registry of acute coronary events scores at admission were divided into three grades: low risk < 109 (31.37%), medium risk 109–140 (38.01%), and high risk > 140 (30.63%) (Table 1). MPO levels were positively correlated with GRACE scores (*r* = 0.22, *p* < 0.001) (Figure 2B), and GRACE scores were higher in patients with high MPO levels than in patients with low and medium MPO levels (*p* = 0.001) (Figure 2A). Considering the correlation between MPO and GRACE scores, we investigated the predictive effect of MPO levels on MACEs in NSTEMI patients with different GRACE risk stratification. When GRACE scores were <140 and ≥140, the number of MACE events in the two groups was 12 and 24, respectively. The prognostic value of mid-to-high MPO level (medium level vs. low level, adjusted HR: 3.65, 95%CI: 1–13.25, *p* = 0.049; high level vs. low level, adjusted HR: 4.688, 95%CI: 1.161–18.928, *P* = 0.03) in GRACE score ≥140 was significantly higher than that in GRACE score <140 (medium level vs. low level, adjusted HR: 0.553, 95%CI: 0.088–3.498, *p* = 0.529; high level vs. low level, adjusted HR: 2.735, 95%CI: 0.42–17.8, *P* = 0.292) (Figure 4C and Supplementary Table 1).

## Discussion

The purpose of this prospective single-center cohort study was to investigate the association of plasma MPO level with GRACE score and prognosis in patients with NSTEMI. The main findings were: first, elevated plasma MPO levels were associated with inflammatory responses, and with increased MPO levels, hs-CRP, leukocyte count, neutrophil count, and fibrinogen levels also increased. Plasma MPO level was also positively correlated with NT-proBNP and hsTNT. Second, with the increase of MPO level, the 1-year incidence of MACEs in patients with NSTEMI was on the rise. Plasma MPO level can be used as an important indicator to predict the occurrence of MACEs in patients with NSTEMI. A subgroup analysis showed that MPO was a powerful prognostic biomarker, and its prognostic value was more significant in patients >65 years and NT-proBNP level >1,000 pg/ml. Finally, plasma MPO levels were positively correlated with GRACE scores, which may contribute to effective risk stratification in patients with NSTEMI. In particular, higher levels of MPO have greater prognostic value in patients classified as high-risk based on GRACE scores.

Myeloperoxidase is a key element of the innate immune system and is released primarily by neutrophils to defend against invading pathogens. During neutrophil activation, the fusion of lysosomes and phagosomes results in the release of MPO, which catalyzes the reaction of hydrogen peroxide (H_2_O_2_) with chloride ion (Cl-) to produce hypochlorous acid (HOCl), helping to destroy microorganisms in the phagocytic lysosomes (15). In “normal” inflammatory conditions (e.g., during infection), active molecules formed in the catalytic cycle are used to eliminate harmful substances, whereas MPO is harmful in chronic clinical or subclinical inflammatory conditions (16). MPO is not only involved in the inflammatory and oxidative stress process of NSTEMI. With the wide application of optical coherence tomography (OCT), more and more studies have confirmed that plaque erosion (PE) is mainly platelet and fibrinogen-based white thrombus, which is an important pathogenesis of NSTEMI (17). The autopsy results showed that a higher level of MPO was detected in the criminal plaque erosion site, and the whole body MPO concentration was also higher in the patients with criminal plaque erosion, indicating that MPO may be related to thrombosis and PE. Further *in vitro* studies have confirmed that MPO can locally produce HOCl, which is an effective oxidant that can induce endothelial cell apoptosis and tissue factor expression, leading to a prethrombotic state, thus promoting the occurrence and development of NSTEMI (18). The elevated level of MPO can provide supplementary information for the diagnosis of plaque types of NSTEMI criminals and achieve more accurate risk stratification and personalized treatment strategies.

In CHD, circulating inflammatory cells (e.g., neutrophils) are a poor prognostic indicator, contributing to the progression of cardiac failure and recurrent coronary events. Thus, identifying markers of inflammation and inflammatory cell activity could help stratify risk in patients with ACS and guide future treatment (19). Early epidemiological studies have assessed the association and clinical usefulness of inflammatory markers such as C-reactive protein, cytokines, white blood cell count, and fibrinogen with CHD, and these inflammatory mediators have been associated with adverse outcomes in patients (20, 21). Kaya et al. showed that elevated plasma MPO level was an independent predictor of MACEs in STEMI patients, and that plasma MPO level was strongly correlated with CRP (*r* = 0.45, *P* = 0.004), but no relationship between MPO and leukocyte count was found. The authors explain that this may be because the study population was small (22). Our study proved that there was a positive correlation between MPO and hs-CRP, leukocyte count, neutrophils, and fibrinogen, reflecting the aggravation of inflammatory state during myocardial infarction. But unlike CRP, MPO is less involved in general systemic inflammation, making it a more specific coronary marker than CRP (23). In addition, MPO activity increases with age (24). In the subgroup analysis of this study, the prognostic value of MPO was more obvious in patients older than 65 years.

Myeloperoxidase can be used as a diagnostic marker for acute CAD. Plasma MPO levels can predict new chest pain early and independently in patients with AMI, even in patients with negative troponin levels, but additional diagnostic information provided by MPO is limited to patients with very early symptoms because MPO secretion occurs early in acute CAD (25). The role of MPO as a biomarker of heart failure has been supported by clinicopathological and epidemiological studies (7). Kaya et al.’s study showed that elevated MPO level was not only an independent predictor of MACEs but also plasma MPO level was positively correlated with NT-proBNP level and negatively correlated with left ventricular systolic function (22). Our study reached a conclusion similar to that of Kaya et al. that baseline MPO levels can predict prognostic information in patients with NSTEMI. In addition, the GUIDing Evidence-Based Therapy Using Biomarker Intensified Treatment in Heart Failure (GUIDE-IT) trial showed that patients with NT-pro BNP declines of ≤1,000 pg/ml during the application of guideline-directed medical therapy (GDMT) have better outcomes. Conversely, the prognosis is worse (26). To this end, we performed a subgroup analysis with 1,000 pg/ml as the cut-off point for NT-pro BNP, and the results showed that for patients with NT-pro BNP >1,000 pg/ml, MPO had a stronger predictive effect on MACEs.

The results of a meta-analysis showed that a high level of MPO significantly predicted the mortality of patients with acute coronary syndrome (OR: 2.03; 95%CI: 1.4–2.94; *P* < 0001), supporting the potential role of MPO as part of a multi-marker risk stratification model to guide future individualized treatment of high-risk populations (19). There is evidence that elevated plasma MPO levels are associated not only with the incidence of CAD but also with the severity of the disease. A case-control study of 874 patients with angiographically confirmed CAD showed elevated MPO levels in patients with CAD and progressively increased MPO levels as CAD stabilization, non-ST-segment elevation acute coronary syndrome, and AMI progressed (27). At the same time, MPO was correlated with the degree of coronary artery stenosis. Heslop et al.’s study showed that plasma MPO levels were higher in patients with severe CAD (defined as stenosis with any lesion confirmed by coronary angiography > 50%) (28). In our study, MPO independently predicted 1-year MACEs in patients with NSTEMI, especially all-cause death and target lesion revascularization. We also found that MPO was positively correlated with GRACE score, especially for patients classified as high risk according to GRACE score, higher level of MPO had greater prognostic value. It suggests that in the future, the plasma MPO level can be used as a part of risk stratification to guide the individualized treatment of high-risk population to reduce the poor prognosis of patients.

Recent studies have pointed to several MPO inhibitors for the prevention and treatment of atherosclerotic cardiovascular disease, such as PF-1355, AZM198, azo radical, and thiocyanate (29). Targeting MPO has the potential to become one of the most promising therapies for coronary heart disease and other atherosclerotic cardiovascular diseases, such as PF-06282999 (8), which has effective and selective MPO inhibition properties, and has been advanced into clinical trials in human pharmacokinetics, safety, tolerance, and MPO inhibition studies (30). However, there are still some challenges to consider in the application of MPO inhibitors. For example, it is not clear whether systemic inhibition of MPO is necessary or whether MPO inhibitors are effective in protecting the heart in patients with an elevated inflammatory environment (29).

This study has some limitations. First, the number of patients was relatively small, which may be one of the reasons why we failed to draw a correlation between MPO and lipids. Second, we failed to dynamically monitor the changes in MPO levels to further understand the individual variability of plasma MPO. Reassessment of plasma MPO levels during follow-up may make our study more useful for prognostic purposes. Finally, given that this was a single-center study with all cases from China, our observations may not be applicable to other clinical settings.

In conclusion, elevated plasma MPO levels are associated with high inflammatory status in patients with NSTEMI. Elevated plasma MPO level is an independent risk factor for prognosis in patients with NSTEMI. The level of MPO is related to GRACE score. A higher level of MPO can independently predict MACEs, especially in patients with a higher GRACE score. It can increase the value of the routine GRACE score of patients with NSTEMI, improve the classification of different risk groups of NSTEMI, and help doctors to give more active treatment. It is recommended that this study be expanded to include large populations of all types of acute coronary syndromes, patients are followed for longer periods of time, markers are dynamically analyzed, and more experimental studies of MPO-targeting inhibitors are needed for early clinical application.

## Data Availability Statement

The original contributions presented in the study are included in the article/Supplementary Material, further inquiries can be directed to the corresponding authors.

## Author Contributions

NZ and J-XW made substantial contributions to the conception, performed the experimental studies, and analyzed the data. JG and YL contributed to the study design. NZ, J-XW, X-YW, YC, and Z-HZ collected the data. NZ, J-XW, JG, and YL prepared the manuscript. All authors read and approved the final version of the manuscript for submission.

## Conflict of Interest

The authors declare that the research was conducted in the absence of any commercial or financial relationships that could be construed as a potential conflict of interest.

## Publisher’s Note

All claims expressed in this article are solely those of the authors and do not necessarily represent those of their affiliated organizations, or those of the publisher, the editors and the reviewers. Any product that may be evaluated in this article, or claim that may be made by its manufacturer, is not guaranteed or endorsed by the publisher.
