# Supplementary material for: Correlation Analysis of Plasma Myeloperoxidase Level With Global Registry of Acute Coronary Events Score and Prognosis in Patients With Acute Non-ST-Segment Elevation Myocardial Infarction
Source: Front Med (Lausanne). 2022 Mar 28;9:828174. doi: 10.3389/fmed.2022.828174 (PMC8995496; doi:10.3389/fmed.2022.828174)
Supplement: Supplementary file 1 [file Data_Sheet_1.docx]

**Supplementary**

Table S1. Relationship between plasma MPO levels and MACEs at 1 year

| Variables | MPO level | HR (95% CI) | P value |
| --- | --- | --- | --- |
| Crude | Low | Reference | – |
|  | Medium | 2.08(0.87,4.93) | 0.098 |
|  | High | 4.53(1.98,10.35) | ＜0.001 |
| Model 1 | Low | Reference | – |
|  | Medium | 2.2(0.87,5.59) | 0.096 |
|  | High | 4.13(1.66,10.29) | 0.002 |
| Model 2 | Low | Reference | – |
|  | Medium | 2.02(0.78,5.19) | 0.146 |
|  | High | 3.25(1.15,9.17) | 0.026 |
| Model 3 | Low | Reference | – |
|  | Medium | 1.92(0.74,4.95) | 0.178 |
|  | High | 3.85(1.4,10.6) | 0.009 |

Model 1: adjustment for traditional cardiovascular risk factors (age, sex, current/former smoking, hypertension, hyperlipidemia, diabetes, previous stroke, and previous MI and family history of CHD).

Model 2: Model 1+ Inflammation Marker (hs-CRP; leukocyte count; neutrophil count; fibrinogen)

Model 3: Model 2+ GRACE Score

Table S2. Subgroup analysis of association between MPO and composite MACEs

| Subgroup | MPO level | Number of events, n (%) | Crude HR,  (95% CI) | Crude  P value | Adjusted HR  (95% CI) * | Adjusted  P value* |
| --- | --- | --- | --- | --- | --- | --- |
| Age≤ 65 | low | 3(33.3%) | Reference | – | Reference | – |
|  | medium | 3(25.0%) | 1.7(0.34,8.42) | 0.516 | 0.47(0.03-7.77) | 0.597 |
|  | high | 2(13.3%) | 2.42(0.4,14.47) | 0.333 | 0.12(0.001-9.46) | 0.338 |
| Age＞ 65 | low | 6(66.7%) | Reference | – | Reference | – |
|  | medium | 9(75.0%) | 2.06(0.73,5.8) | 0.169 | 2.53(0.79,8.09) | 0.118 |
|  | high | 13(86.7%) | 4.2(1.59,11.06) | 0.004 | 5.7(1.54,21.05) | 0.009 |
| NT-proBNP ≤1000 | low | 3(42.9%) | Reference | – | Reference | – |
|  | medium | 2(16.7%) | 1.03(0.17,6.17) | 0.973 | 1.15(0.16,8.14) | 0.163 |
|  | high | 2(20.0%) | 4.35(0.88,21.55) | 0.072 | 1.98(0.27,14.73) | 0.267 |
| NT-proBNP ＞1000 | low | 4(57.1%) | Reference | – | Reference | – |
|  | medium | 10(83.3%) | 3.65(1.14,11.64) | 0.029 | 6.26(1.56,25.12) | 0.01 |
|  | high | 12(80.0%) | 5.19(1.67,16.1) | 0.004 | 7.69(1.75,33.71) | 0.007 |
| GRACE＜140 | low | 5(53.3%) | Reference | – | Reference | – |
|  | medium | 3(33.2%) | 0.96(0.23,4.01) | 0.95 | 0.553(0.088,3.498) | 0.529 |
|  | high | 4(13.6%) | 3.48(0.93,12.95) | 0.06 | 2.735(0.42,17.8) | 0.292 |
| GRACE ≥140 | low | 4(44.4%) | Reference | – | Reference | – |
|  | medium | 9(75.0%) | 3.18(0.98,10.32) | 0.055 | 3.65(1.0,13.25) | 0.049 |
|  | high | 11(73.3%) | 3.35(1.06,10.52) | 0.039 | 4.688(1.161,18.928) | 0.03 |


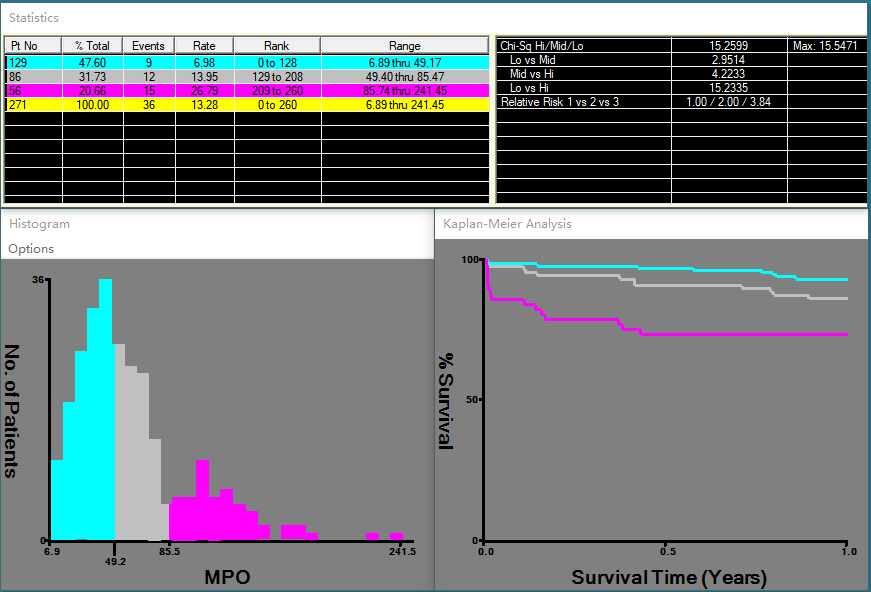


Figure S1：The MPO cut-off point selected according to the X-tile software
